# Endolymphatic Potential Measured From Developing and Adult Mouse Inner Ear

**DOI:** 10.3389/fncel.2020.584928

**Published:** 2020-12-07

**Authors:** Yi Li, Huizhan Liu, Xiaochang Zhao, David Z. He

**Affiliations:** ^1^Department of Otorhinolaryngology-Head and Neck Surgery, Beijing Tongren Hospital, Capital Medical University, Beijing, China; ^2^Department of Biomedical Sciences, Creighton University School of Medicine, Omaha, NE, United States

**Keywords:** cochlea, vestibule, endolymphatic potential, endocochlear potential, vestibular microphonic, mouse

## Abstract

The mammalian inner ear has two major parts, the cochlea is responsible for hearing and the vestibular organ is responsible for balance. The cochlea and vestibular organs are connected by a series of canals in the temporal bone and two distinct extracellular fluids, endolymph and perilymph, fill different compartments of the inner ear. Stereocilia of mechanosensitive hair cells in the cochlea and vestibular end organs are bathed in the endolymph, which contains high K^+^ ions and possesses a positive potential termed endolymphatic potential (ELP). Compartmentalization of the fluids provides an electrochemical gradient for hair cell mechanotransduction. In this study, we measured ELP from adult and neonatal C57BL/6J mice to determine how ELP varies and develops in the cochlear and vestibular endolymph. We measured ELP and vestibular microphonic response from saccules of neonatal mice to determine when vestibular function is mature. We show that ELP varies considerably in the cochlear and vestibular endolymph of adult mice, ranging from +95 mV in the basal turn to +87 mV in the apical turn of the cochlea, +9 mV in the saccule and utricle, and +3 mV in the semicircular canal. This suggests that ELP is indeed a local potential, despite the fact that endolymph composition is similar. We further show that vestibular ELP reaches adult-like magnitude around post-natal day 6, ~12 days earlier than maturation of cochlear ELP (i.e., endocochlear potential). Maturation of vestibular ELP coincides with the maturation of vestibular microphonic response recorded from the saccular macula, suggesting that maturation of vestibular function occurs much earlier than maturation of hearing in mice.

## Introduction

The inner ear of mammals has two major parts, the cochlea is responsible for hearing and the vestibular organ is responsible for balance. The cochlea and vestibular organ are connected by a series of canals in the temporal bone referred to as the bony labyrinth. The bone canals are separated by the membranes in parallel spaces referred to as the membranous labyrinth. Two distinct extracellular fluids, endolymph and perilymph, separated by a membranous labyrinth, fill different compartments of the inner ear. Compartmentalization of the fluids establishes an electrochemical gradient between the endolymph, perilymph, and the intracellular compartments necessary for hair cell transduction. Perilymph is found in the scala tympani and scala vestibuli in the cochlea. The perilymph, in continuity with cerebrospinal fluid of the subarachnoid space via the cochlear aqueduct, is rich in Na^+^ ions and low in K^+^ ions. Endolymph is the fluid contained within the scala media of the membranous labyrinth of the inner ear; endolymph is high in K^+^ ions and resembles intracellular fluid in its composition. The endolymph in the scala media continues beyond the base of the cochlea into the vestibular portion of the inner ear. The scala media connects to the saccule via the ductus reuniens, and the endolymph reaches utricle and semicircular canals through the utriculo-endolymphatic duct. The stria vascularis and vestibular dark cells and transitional cells are responsible for endolymph secretion. These structures and cells are the foundations for regulation of inner-ear homeostasis. Impaired function of these structures due to mutations, damage, and aging leads to hearing loss (Steel and Barkway, 1989; Wu and Marcus, 2003; Gow et al., 2004; Wangemann et al., 2004; Ohlemiller et al., 2006; Cohen-Salmon et al., 2007; Sha et al., 2008; Ni et al., 2013; Zhang et al., 2014; Liu et al., 2016).

The endolymph within the scala media exhibits a constant ~80-mV positive polarization with respect to the perilymph (Von Bekesy, 1952). This high positive potential in endolymph is called endocochlear potential (EP) or endolymphatic potential (ELP). Because ELP is present in the cochlear and vestibular endolymph, we use EP and vestibular ELP (vELP), respectively, to describe ELP measured from the cochlear and vestibular endolymph. ELP and the concentration gradient of K^+^ and Ca^2+^ ions between the endolymph and cytosol of hair cells are thought to be the electrochemical driving force for hair cell mechanotransduction currents (Davis, 1957, 1965).

ELP has been measured from the auditory and vestibular endolymphatic compartments of mammals and non-mammals (Köppl et al., 2018). While EP is ~+80 mV in mammals, vELP ranges from +1 to +11 mV (Tasaki, 1957; Misrahy et al., 1958; Smith et al., 1958; Schmidt and Fernandez, 1962; Schmidt, 1963; Ninoyu et al., 1987; Hommerich, 1990). Since most previous measurements were not obtained from the same species in a similar experimental setting, it is unclear how ELP varies in the inner ear endolymph. Furthermore, development of vELP has not been examined, despite extensive examination into development of EP in the cochleae of mice, gerbils, guinea pigs, cats, and pigs (Schmidt and Fernandez, 1963; Bosher and Warren, 1971; Fernández and Hinojosa, 1974; Uziel et al., 1981; Woolf et al., 1986; Ohmura and Yamamoto, 1990; Rybak et al., 1992; McGuirt et al., 1995; Sadanaga and Morimitsu, 1995; Guo et al., 2017). Thus, it is still unknown how vELP in different vestibular end organs develops and when it matures. In this study, we measured ELP from adult C57BL/6J mice to determine how ELP varies in the endolymphatic space of the cochlea and vestibule. We also measured development of EP and vELP as well as development of vestibular microphonic response from saccular macula to determine when ELP and vestibular microphonic are functionally mature. These measurements allow us to directly compare the magnitude of ELP recorded from the cochlea and vestibule in adult and neonatal mice. Our measurements from adult mice shows that ELP varies significantly in the inner ear, despite the fact that endolymph ionic composition is similar in the cochlea and vestibule. We further show that vELP and vestibular microphonic reach adult-like responses around post-natal day 6 (P6), suggesting that maturation of vELP and vestibular function occurs much earlier than maturation of EP and auditory function in mice.

## Materials and Methods

### Animals

Neonatal and adult C57BL/6J mice were bred in-house. For electrophysiological experiments, mice were anesthetized with a combined regimen of ketamine (16.6 mg/ml) and xylazine (2.3 mg/ml) and supplemented as needed to maintain a surgical level of anesthesia. Care and use of the animals in this study were approved by the Institutional Animal Care and Use Committees of Capital Medical University (Beijing) and Creighton University as well as grants from National Science Foundation of China (#81600798 and #81770996) and NIH (R01 DC016807) from the NIDCD.

### Recording of EP Through the Lateral Wall of the Cochlea

After anesthesia, tracheotomy was performed in the ventral position without artificial respiration. The tympanic bulla was opened after tissue and musculature overlying the bulla were carefully removed. With a clear view of the cochlea, a small hole was made in the lateral wall of the cochlea in the apical or basal turn using a fine drill. A glass capillary microelectrode (5–8 MΩ) filled with 150 mM KCl was mounted on a Leica micromanipulator. The ground electrode was implanted in the dorsal neck muscles. The microelectrode was inserted into the scala media through the spiral ligament and the stria vascularis by controlling movement of the micromanipulator. The response from the microelectrode was amplified (high-pass filtered at 1 kHz) under current-clamp mode using an Axopatch 200B amplifier (Molecular Probe, Sunnyvale, CA, USA) and acquired by software pClamp 10 (Molecular Devices) running on an IBM-compatible computer with a 16-bit A/D converter (Digidata 1440A). The voltage changes during a penetration were continuously recorded under the gap-free model using the Clampex in the pClamp software package (version 10, Molecular Devices). The sampling frequency was 5 kHz. Data were analyzed using Clampfit and Excel.

### Recording ELP From Cochlea, Utricle, Saccule, and Semicircular Canal Through Round Window

The microelectrode was mounted on a Leica micromanipulator. The electrode, placed under visual control onto the round window, was advanced manually toward the organ of Corti through the round window membrane. The baseline was adjusted to zero with the tip of the microelectrode in the scala tympani. The microelectrode was then advanced across the scala tympani, through the region of the organ of Corti, and finally into scala media. When the microelectrode passed through the region of the organ of Corti, a negative DC potential was recorded. A stable positive DC potential (i.e., the EP) was observed when the micropipette entered the scala media.

To record vELP from utricle or saccule, the microelectrode advanced further from the scala media to scala vestibuli and finally to the saccule or utricle. To record vELP from the semicircular canal, a small hole was drilled in the anterior canal. The microelectrode, under the control of Leica micromanipulator, was advanced through the membrane labyrinth until a stable positive potential was recorded. To confirm that the recording was made from utricle or saccule, we used fluorescent phalloidin (Lot #: 565227, Invitrogen) to stain F-actin (cytoskeleton and stereocilia bundles of vestibular hair cells). The tip of the microelectrode contained fluorescent phalloidin diluted in 150 mM KCl (with an estimated concentration of 1:100). Phalloidin was injected into the saccule or utricle by applying a positive pressure to the microelectrode. After vELP was measured, the utricle and saccule were dissected out and fixed in 4% PFA for 15 min. The location of the fluorescent stain was determined under a confocal microscope (Zeiss LSM 710).

### *In vivo* Recording of Vestibular Microphonic From Saccular Maculae

The vestibular microphonic was recorded using the same microelectrode to record vELP. To evoke vestibular microphonic from the macula of saccule, direct stapes driving by a piezo actuator was used (Woolf and Ryan, 1988). The stimulus used to evoke vestibular microphonic was a 400 Hz sinusoid with duration of 100 ms and rise-time of 1 ms. The stimulus was fed to a Burleigh PZ-150M Driver. The piezo actuator (transducer) was glued to the stapes foot through a tapered glass rod (1.5 cm in length and 0.6 mm in diameter at the stapes foot) after the ossicular chain was severed. The magnitude of the piezo motion (peak-to-peak) was pre-set at five levels from ~1 to 3 μm in 0.5 μm increment. We did not expect contribution of cochlear microphonic to our recording as the frequency range of the basilar membrane response (or hearing frequency) of C57BL/6 is above 2 kHz. To rule out the possibility of contribution by cochlear microphonic at high stimulus level, the apical turn of the cochlea was destroyed before vestibular microphonic was recorded. The microphonic signal from the microelectrode was amplified and high-pass filtered (at 1 kHz) under current-clamp mode using an Axopatch 200B amplifier and acquired by software pClamp 10 running on an IBM-compatible computer with a 16-bit A/D converter (Digidata 1440A). The sampling frequency was 5 kHz. Response from 20 presentations was averaged for each recording.

### Statistical Analysis

Means and standard deviations (SD) were calculated based on measurements from eight mice at each condition, location and age. Student's *t*-test was used to determine statistical significance between two different conditions/locations. Probability (P) value ≤ 0.01 was regarded as significant.

## Results

### EP From Apical and Basal Cochlear Turns of Adult Mice

**Figure 1:**
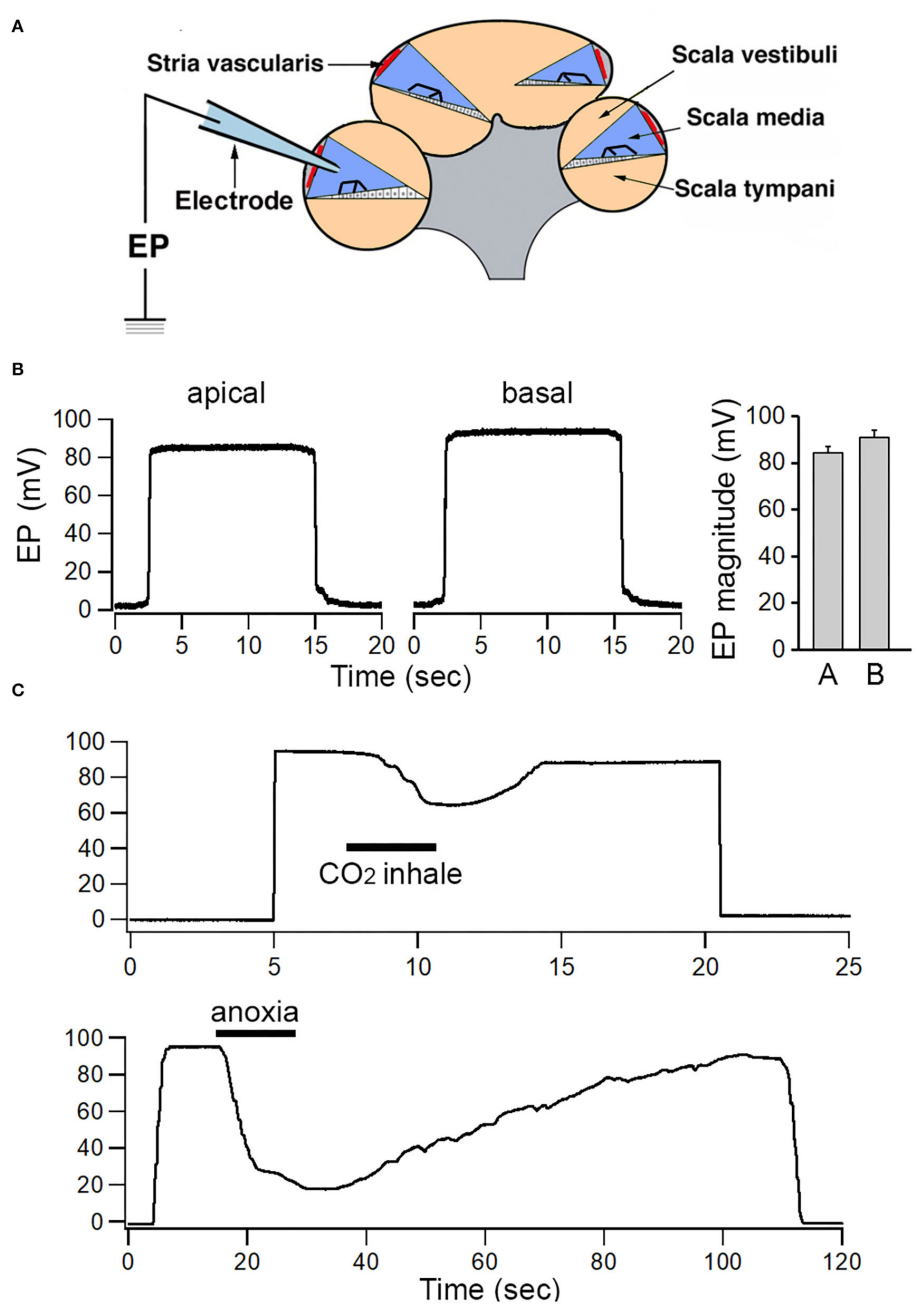
Recording of EP from apical and basal turns of the cochlea. **(A)** Schematic view of electrode path for recording EP through lateral wall approach. **(B)** Examples of EP recorded from apical and basal turns. Mean and SD of EP recorded from eight apical and eight basal turn locations are shown in the right panel. **(C)** Change in EP magnitude in response to temporal CO_2_ inhalation and anoxia.

EP has been measured by using microelectrode through the lateral wall (i.e., spiral ligament and stria) of the cochlea or through the round window, scala tympani and the basilar membrane. Since the round window approach only allows measurement of EP in the basal turn, the lateral wall approach (Figure 1A) was used so that EP from apical and basal turns could be measured and compared (Zhang et al., 2014; Liu et al., 2016). Figure 1B shows some examples of EP measured from the apical and basal turns of a one-month-old mouse. The mean EP magnitude obtained from the apical and basal turns of eight mice is presented in the right panel of Figure 1B. The mean EP magnitude measured from the basal turn is +92.3 mV. The EP measured from apical turn is ~7 mV less than that of the basal turn, with a mean magnitude of +85.8 mV. The difference is statistically significant (*p* < 0.01, *n* = 8).

As EP is highly dependent on metabolism (Konishi and Teruzo, 1961; Schmidt and Fernandez, 1962; Woolf et al., 1986; Liu et al., 2016), we examined change in EP magnitude during brief inhalation of CO_2_ or brief anoxia. CO_2_ was applied to the area near the opening in the trachea. To examine short-term effects of anoxia on ELP, the trachea was temporally occluded by a pair of forceps. As shown in Figure 1C, the magnitude of EP drops from ~30 to 50 mV within a few seconds after CO_2_ inhalation or after oxygen intake was blocked. The reduction was reversible after CO_2_ inhalation or anoxia was ceased.

### Development of EP

**Figure 2:**
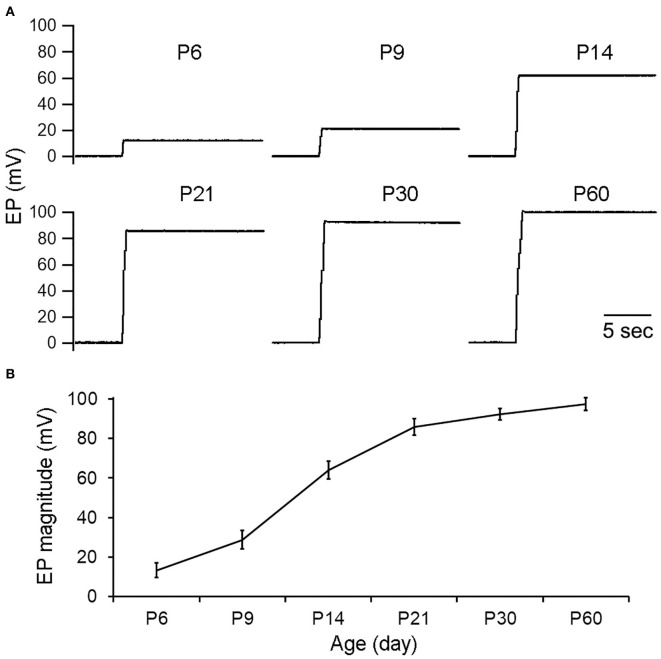
EP during development. **(A)** Representative examples of EP recorded from cochlear basal turn during development. Age is shown on top of each recording/panel. **(B)** Mean and SD of EP during development (*n* = 8 for each age group).

We measured EP from the cochlear basal turn during development using mice aged between P6 and P60. Figure 2 shows some examples of EP recorded at P6, P9, P14, P21, P30, and P60. Eight mice at each age were measured and mean magnitude of EP is presented in Figure 2B. The magnitude of EP was about +16 mV at P6. At P14, 2 days after hearing onset in mice, the magnitude was >+60 mV. The magnitude continued to grow after P14 and reached +80 mV at P21. The EP magnitude grew again between P21 and P60, although at much slower rate. At P60, the magnitude was >+95 mV. This is consistent with slow EP growth seen between P20 and P60 in previous studies in gerbils (McGuirt et al., 1995) and C57BL/6J mice (Zhang et al., 2014).

### ELP and Its Development in Saccule and Utricle

**Figure 3:**
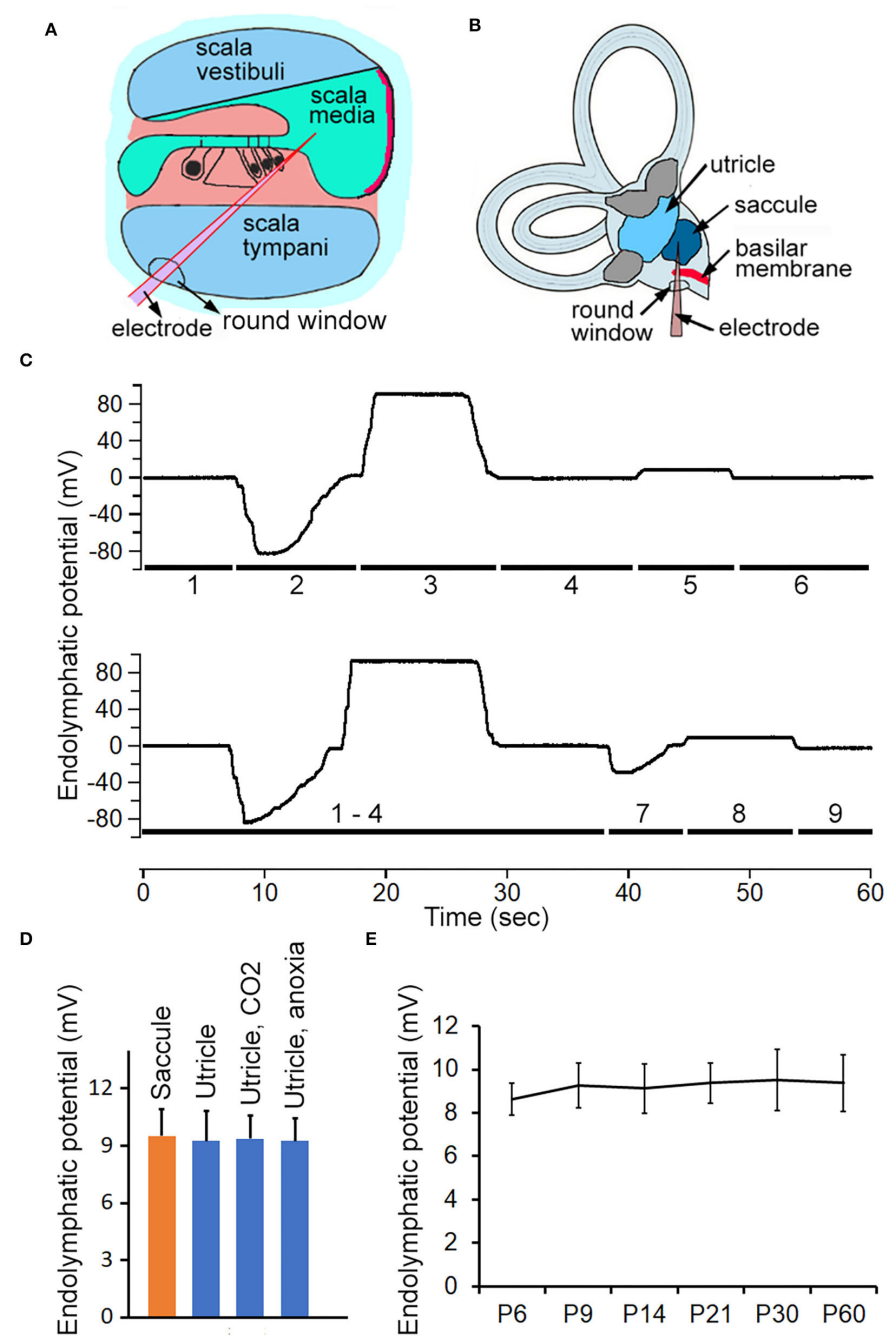
Recording ELP from cochlea, saccule and utricle in adult and neonatal mice. **(A)** Schematic view of electrode path for recording EP through round window approach. **(B)** Electrode path for recording ELP from saccule through round window. **(C)** Representative examples of changes in recorded potential as micropipette advancing from scala tympani, through basilar membrane, and into scala media and scala vestibuli, and finally into saccule or utricle. The number underneath the response indicates the location where the response was recorded. 1: scala tympani. 2: region of organ of Corti. 3: scala media. 4: scala vestibuli. 5: saccule. 6: scala vestibuli. 7: utricular macula. 8: utricle. 9: withdrawing the electrode (into scala vestibuli would return the potential to zero or a slightly negative potential. **(D)** Mean magnitude and SD of ELP recorded from saccule and utricle (*n* = 8 for either). Utricular ELP magnitude after inhalation of CO_2_ and anoxia is also presented. No statistical significance was found among them. **(E)** Development of ELP of saccule. Mean and SD are presented (*n* = 8).

ELP from different vestibular end organs was measured to determine if its magnitude was substantially different from EP. To measure ELP from saccule and utricle, the round-window approach was used (Figures 3A,B). Representative examples of recorded potentials are exhibited in Figure 3C as a microelectrode was advanced from scala tympani through the region of the organ of Corti, and into scala media, scala vestibuli, and finally into saccule or saccule. As shown, a negative potential of 80 to 85 mV is seen when the microelectrode passed through the regions of the organ of Corti (Woolf et al., 1986). This negative organ of Corti potential lasted for a short time, then decayed back to zero. Further advancement of the micropipette into scala media resulted in recording of a stable positive DC potential of 90 to 95 mV: the positive EP. The potential went back to zero after the electrode entered scala vestibuli. After the microelectrode passed through the lateral side of the sac of saccule, a stable positive potential of 8–10 mV was seen. When the microelectrode was directed toward utricle after entering the scala vestibuli, a negative potential of ~30 to 40 mV was observed. This potential is similar to the negative potential seen during electrode penetration through part of the organ of the Corti, except that the magnitude of the potential is substantially less. After penetrating through the macula of utricle, a stable positive potential of 8–10 is observed. This is the ELP of the utricle. We recorded vELP from saccule and utricle from eight adult mice. The mean magnitude was 9.5 ± 1.4 mV for saccule and 9.3 ± 1.6 mV for utricle (Figure 3D). No statistical significance was found between them. We measured utricular ELP to determine if utricular ELP is also sensitive to inhalation of CO_2_ and anoxia. As shown in Figure 3D, the magnitude of ELP is not significantly reduced in response to CO_2_ inhalation or anoxia.

To determine how vELP changes during development, vELP was measured from saccule of neonatal mice aged between P6 and P60. The mean magnitude is presented in Figure 3E. As shown, the magnitude of vELP from saccule at P6 is not statistically different from that measured at later stages during development, suggesting that vELP already reaches adult-like response at P6.

**Figure 4:**
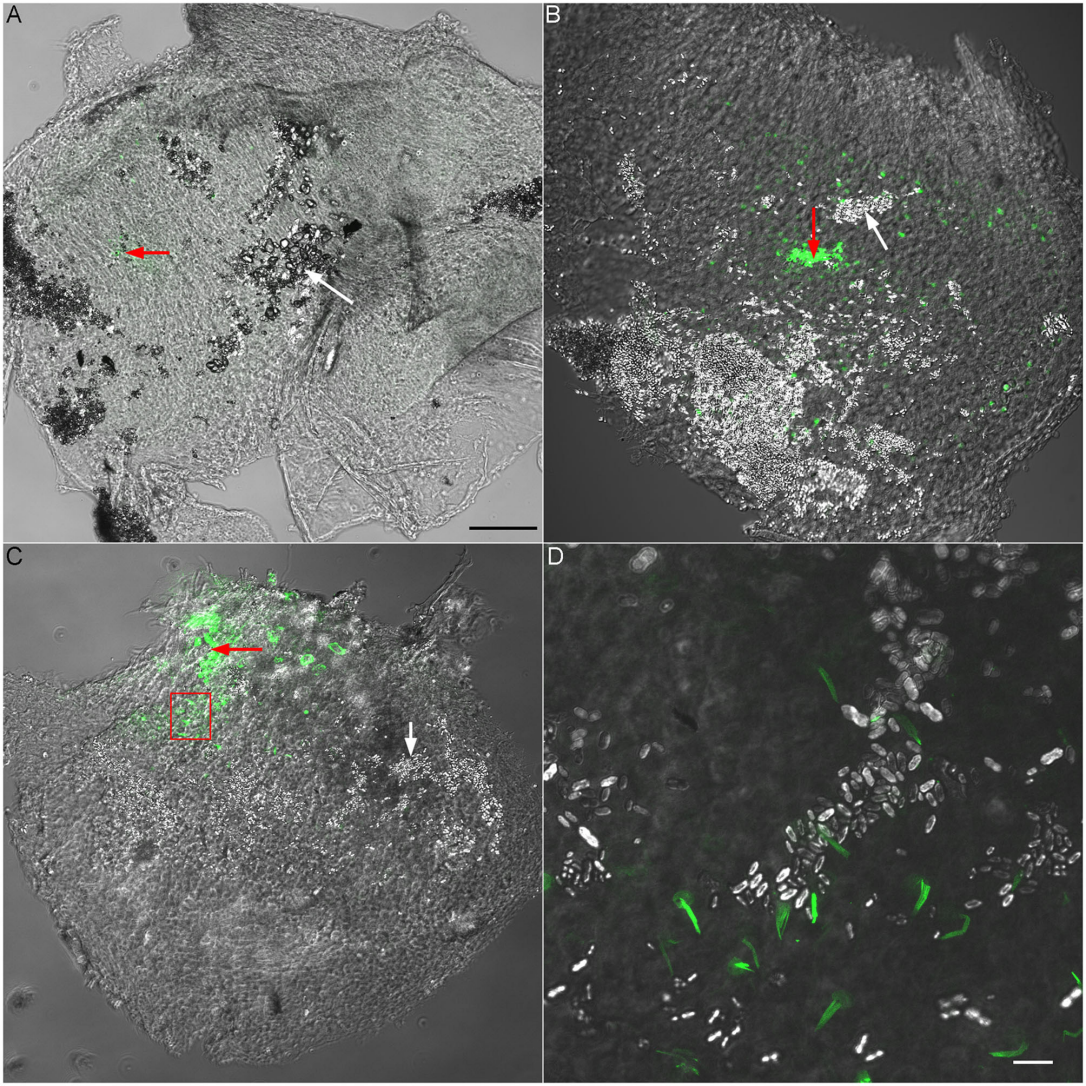
Validation of recording site using fluorescent phalloidin. **(A)** Superimposed confocal and DIC images (10 × lens) obtained from a P6 utricle. Fluorescent phalloidin was injected to the recording site by applying a positive pressure to the recording electrode after ELP was recorded. The area with fluorescent signal is marked by a red arrow. A white arrow marks otoconia in a nearby area. Scale bar: 100 μm [for **(A)**, **(B)** and **(C)** panels]. **(B)** Superimposed confocal and DIC images of a P6 saccule. A red arrow marks the area with fluorescent signal (recording site) while a white arrow marks otoconia. **(C)** Superimposed confocal and DIC images from a P30 saccule. The area with a frame is presented in panel D with higher magnification. **(D)** Superimposed confocal and DIC images (63 × lens) of phalloidin-labeled stereocilia bundles of saccular hair cells. Otoconia can also been seen. Scala bar: 10 μm.

To verify that the recording electrode was indeed in utricle or saccule, we used fluorescent-labeled phalloidin to stain F-actin. Fluorescent phalloidin was injected to the location of recording by applying positive pressure to the microelectrode after vELP was measured. Since phalloidin stained stereocilia bundles and cytoskeleton of hair cells in the area near the tip of the microelectrode, the location of measurement could be confirmed. Figure 4 shows some representative images obtained from neonatal (P6) and adult saccule and utricle. As shown, the fluorescent signal is observed in a small area in utricle or saccule. Under higher magnification, stereocilia bundles are clearly visible (Figure 4D), confirming that our recordings were made from utricle and saccule.

### *In vivo* Recording of Vestibular Microphonic From the Macula of Saccule

**Figure 5:**
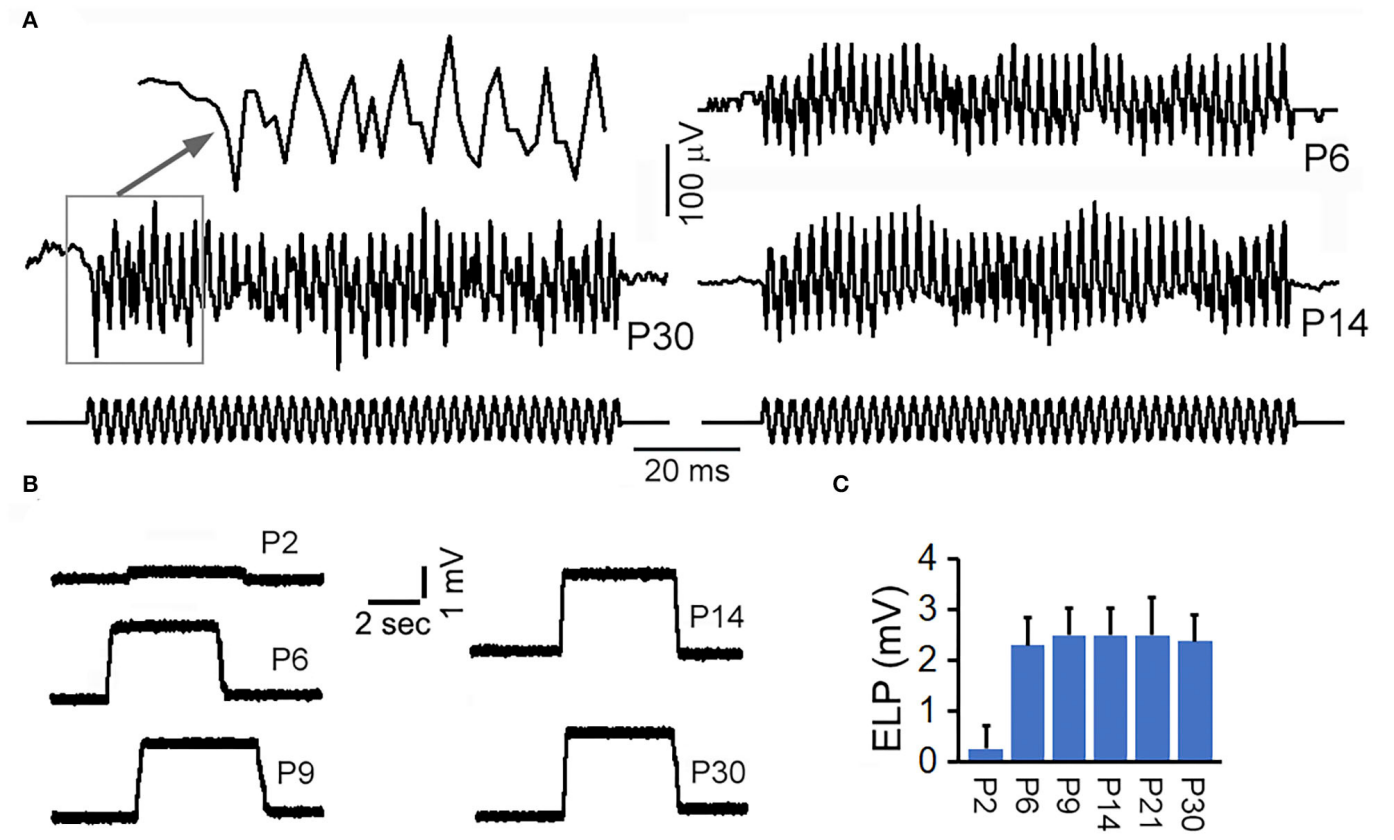
Vestibular microphonic recorded from saccular macula and ELP recorded from semicircular canal. **(A)** Vestibular microphonic recorded from saccular maculae of neonatal and adult mice. **(B)** ELP recorded from semicircular canal of neonatal and adult mice. **(C)** Mean and SD of ELP from semicircular canal at different ages.

Trincker (Trincker, 1959) used sharp microelectrode technique to measure vestibular microphonic in response to air-conducted sound to stimulate the vestibular system following surgical destruction of the cochlea, whereas Pastras et al. (2017) recorded vestibular microphonic in response to bone-conducted vibration from utricular or saccular macula. We measured microphonic from saccular maculae from adult and neonatal mice by directly driving stapes motion to determine when saccule was functionally mature during development. Large stimulus was used to drive the response to saturation. The response magnitude at different time points during development was compared. Figure 5A shows some representative response waveforms obtained from the saccular maculae of P6, P14, and P30 mice. As shown, the vestibular microphonic resembles the stimulus with strong distortion due to higher stimulation level. The peak-to-peak magnitude of the saturated response at P30 is ~200 μV. This magnitude was comparable to a previous study using bone-conducted vibration (Pastras et al., 2017). We compared the peak-to-peak magnitude of the saturated response obtained from P6, P14, and P30 mice. The magnitude at P6 was not statistically different from that at P14 and P30, suggesting that vestibular microphonic is already adult-like at P6.

### ELP and Its Development in the Semicircular Canal

ELP from anterior semicircular canal was measured in adult and neonatal mice. After the electrode penetrated through the membrane labyrinth, a stable positive potential of ~2 to 3 mV was observed (Figure 5B). This DC potential was substantially smaller than ELP recorded from saccule and utricle. We measured vELP from semicircular canal of eight mice at P30 and the mean magnitude was +2.5 ± 0.8 mV (bottom panel of Figure 5C). To determine how vELP develops in the semicircular canal, neonatal mice aged between P2 and P30 were used. As shown in Figure 5C, vELP is small at P2, < +0.4 mV. At P6, the magnitude reaches 2.2 mV, which is not statistically significant from the magnitude observed at P9 and other later stages during development. This suggests that the ELP in the semicircular canal reaches adult-like response before or at P6.

## Discussion

**Figure 6:**
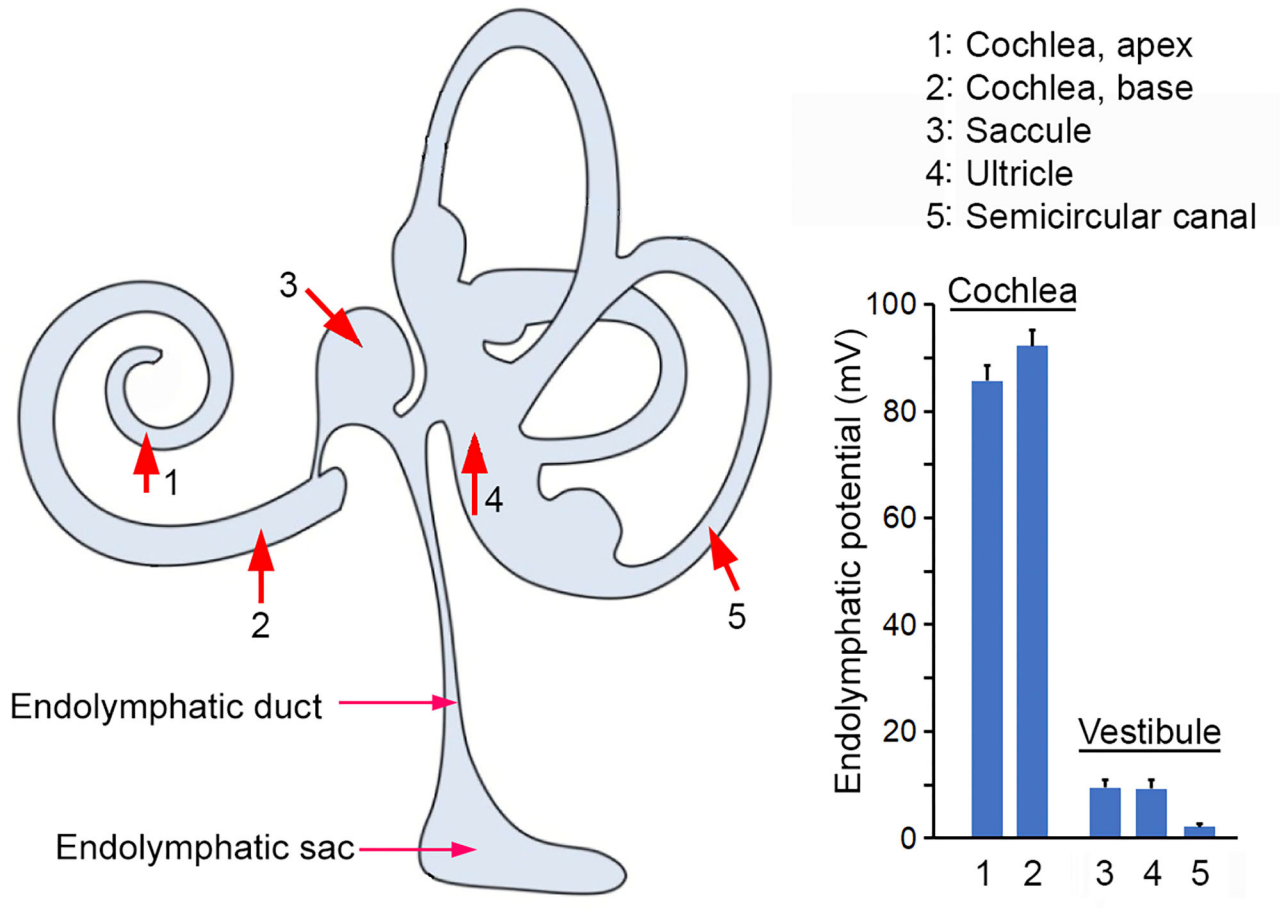
ELP recorded from the inner ear. Red arrows and numerical numbers mark the location where ELP was measured. The magnitude of ELP at different endolymphatic compartments in the inner ear is shown in the right panel. Schematic drawing in the left panel was modified from Kim et al. (2011) with permission.

By measuring ELP from endolymphatic space in different inner ear compartments of adult mice, we showed that the magnitude of ELP maintained in endolymph relative to perilymph varies significantly, ranging from +95 mV in the cochlear base and +87 mV in the cochlear apex, to +9 mV in the maculae of saccule and utricle, to +3 mV in the semicircular canal. The magnitude of ELP at different locations of the cochlear and vestibular endolymphatic space is summarized in Figure 6. In the cochlea, the difference between apical and basal turn is ~8 mV. This is consistent with 7 mV seen in mice (Sadanaga and Morimitsu, 1995) and close to ~10 mV observed in guinea pigs and rats (Kuijpers and Bonting, 1970; Rybak et al., 1992). It is not clear why EP is different between the apex and base. We speculate that more stria cells in the cochlear base may contribute to a larger EP since the size of stria is bigger in the basal turn of the cochlea and since EP is generated and maintained by stria.

Our study also showed that ELP in the vestibular endolymphatic compartment is between +3 to +9 mV. Previous studies in guinea pigs showed that vELP ranges from +1 to +11 mV. Tasaki (Tasaki, 1957) was the first to show that the potential in the vestibular endolymph is nowhere near as high as in the cochlear endolymph. Subsequent studies by Misrahy et al. (1958) and by Smith et al. (1958) reported a potential of no more than +5 mV in the guinea pig utricle and of +1 mV in the saccule. Similar lower values were observed in several different non-mammalian species (Schmidt and Fernandez, 1962). A vELP value of +3 to +9 mV observed in the present study is similar to the value reported in previous studies. The reason of why ELP is much lower in the vestibular part of the endolymph is not known. While the EP is generated and maintained by stria vascularis in the cochlea, it is less clear what structure and cell types are involved in ELP generation and maintenance in the vestibular endolymph. Some studies suggest that dark cells and transitional cells are involved (Köppl et al., 2018). It is conceivable that the difference in structure and cell types is responsible for the difference. We note that while EP is highly dependent on metabolism and that anoxia leads to a significant reduction of EP (Figure 1C), ELP recorded from saccule seems to be not sensitive to brief anoxia and CO_2_ poisoning (Figure 3D). It is not clear why vLEP is not sensitive to anoxia. But we note that the EP of all ectothermic species such as turtles, snakes and lizards is also not sensitive to hypoxia (Schmidt and Fernandez, 1962; Schmidt, 1963). This does not suggest the absence of any electrogenic component to the EP in mouse utricle or in those species. It may reflect lower metabolic rates and/or an extracellular environment in which those cells live and function.

The difference in ELP magnitude between the cochlear and vestibular endolymph compartments suggests that ELP is a local potential with limited capability to spread (e.g., +95 mV in the basal turn of the cochlea vs. +8 mV in saccule), perhaps due to the leak current of the mechanotransduction channels in the stereocilia of hair cells. It is unclear why a larger electrical gradient is needed for cochlear hair cells but not so much for vestibular hair cells. However, it is clear that the ELP magnitude is not directly proportional to superiority of high sensitivity as non-mammalian vertebrates such as birds, which exhibit comparable sensitivity as mammals, show a lower EP between 0 and +35 mV. This is closer to the potential observed in the vestibular endolymph of the mammalian inner ear, despite the fact that the ionic composition is similar in all vertebrates and in vestibular and cochlear endolymph. The huge difference of ELP in cochlear and vestibular end organs does raise two important questions: Is the magnitude of EP really critical for mechanotransduction and why ELP needs to be substantially higher in the cochlea than in the vestibular end organs? The results from current study seem to suggest that the magnitude of ELP may not be as critical as believed; hair cells in saccule, utricle and crista ampullaris do not seem to rely on a larger driving force (electrical gradient) for mechanotransduction.

While cochlear microphonic has extensively been used to examine hair cell and cochlear function, vestibular microphonic has only been reported in a handful of publications mostly involving non-mammalian and *ex vivo* models (Adrian et al., 1938; Zotterman, 1943; Lowenstein and Roberts, 1951; Corey and Hudspeth, 1983; Rabbitt et al., 2005). Trincker (1959) used air-conducted sound to stimulate the vestibular system, and maintained fluid within the vestibule following surgical destruction of the cochlea, whereas Pastras et al. (2017) measured vestibular microphonic from utricular or saccular macula using bone-conducted vibration. We measured vestibular microphonic from developing and adult mice. The waveform and magnitude of microphonic response recorded from saccular maculae of adult mice were similar to those observed in the utricular or saccular maculae of adult guinea pigs (Pastras et al., 2017). We note that the microphonic response recorded was likely from a combined hair cell and neural response. The development of vestibular microphonic has not been examined in neonatal animals. Our measurement showed, for the first time, that the magnitude of vestibular microphonic recorded from the saccular macula was adult-like as early as P6, coinciding with maturation of vELP.

Development of EP has been examined in rabbits, rodents, guinea pigs and pigs before. In rabbit, EP development begins at P5 and reaches adult-like magnitude around P15 (Anggard, 1965). In rodents such as rat, gerbil, and mouse, EP development occurs between P8 and P20 (Bosher and Warren, 1971; Woolf et al., 1986; Rybak et al., 1992; McGuirt et al., 1995; Sadanaga and Morimitsu, 1995). Sadanaga and Morimitsu (1995) measured EP from apical and basal turns of neonatal mice, reporting an EP of <10 mV before P8, ~20 mV at P9, ~50 mV at P12 and ~70 mV at P13, finally reaching an adult-like magnitude at P20. This time course of EP development is very similar to that seen in gerbils (Woolf et al., 1986; McGuirt et al., 1995). We measured EP from the basal turn during development and showed a similar time-course of maturation as reported by others. In contrast to the time-course of EP maturation, our measurement showed that vELP development in two locations in the vestibular part of endolymph occurs much earlier; the magnitude reached adult-like level near P6, ~12 days earlier than maturation of EP. As far as we know, development of ELP in the vestibular endolymph has not been examined before. The fact that vELP is adult-like at P6 suggests that vestibular dark cells and transitional cells, which are assumed to be responsible for generating and maintaining ELP in the vestibular end organs (Wangemann, 1995), are functional mature by P6. Maturation of vELP coincides with maturation of vestibular microphonic observed in the saccular macula during development, suggesting that maturation of vELP and vestibular function occurs much earlier than maturation of EP and auditory function in mice. This is consistent with behavioral evaluation which shows that surface righting reflex that echoes labyrinth and postural response is adult-like between P6 and P8 (Castelhano-Carlos et al., 2010).

## Data Availability Statement

The raw data supporting the conclusions of this article will be made available by the authors, without undue reservation.

## Ethics Statement

The animal study was reviewed and approved by Capital Medical University, Beijing, China.

## Author Contributions

YL and DH designed the experiments. YL, HL, XZ, and DH performed the experiments. YL and DH wrote the manuscript. All authors contributed to the article and approved the submitted version.

## Conflict of Interest

The authors declare that the research was conducted in the absence of any commercial or financial relationships that could be construed as a potential conflict of interest.
